# Advances in Immunotherapy for Melanoma: A Comprehensive Review

**DOI:** 10.1155/2017/3264217

**Published:** 2017-08-01

**Authors:** Carmen Rodríguez-Cerdeira, Miguel Carnero Gregorio, Adriana López-Barcenas, Elena Sánchez-Blanco, Beatriz Sánchez-Blanco, Gabriella Fabbrocini, Brunilda Bardhi, Ardiana Sinani, Roberto Arenas Guzman

**Affiliations:** ^1^Dermatology Service, Hospital do Meixoeiro and University of Vigo, Vigo, Spain; ^2^Department of Biochemistry, Genetics and Immunology, University of Vigo, Vigo, Spain; ^3^Mycology Service, Hospital Manuel Gea González, Mexico City, Mexico; ^4^Health Department, Xunta Galicia, Vigo, Spain; ^5^Galician Healthcare Service, Vigo, Spain; ^6^Dermatology Service, University of Napoli Federico II, Naples, Italy; ^7^Dermatology Service, Venus Clinic, Tirana, Albania; ^8^Dermatology Service, Military Medical Unit, University Trauma Hospital, Tirana, Albania

## Abstract

Melanomas are tumors originating from melanocytes and tend to show early metastasis secondary to the loss of cellular adhesion in the primary tumor, resulting in high mortality rates. Cancer-specific active immunotherapy is an experimental form of treatment that stimulates the immune system to recognize antigens on the surface of cancer cells. Current experimental approaches in immunotherapy include vaccines, biochemotherapy, and the transfer of adoptive T cells and dendritic cells. Several types of vaccines, including peptide, viral, and dendritic cell vaccines, are currently under investigation for the treatment of melanoma. These treatments have the same goal as drugs that are already used to stimulate the proliferation of T lymphocytes in order to destroy tumor cells; however, immunotherapies aim to selectively attack the tumor cells of each patient. In this comprehensive review, we describe recent advancements in the development of immunotherapies for melanoma, with a specific focus on the identification of neoantigens for the prediction of their elicited immune responses. This review is expected to provide important insights into the future of immunotherapy for melanoma.

## 1. Introduction

**Figure 1:**
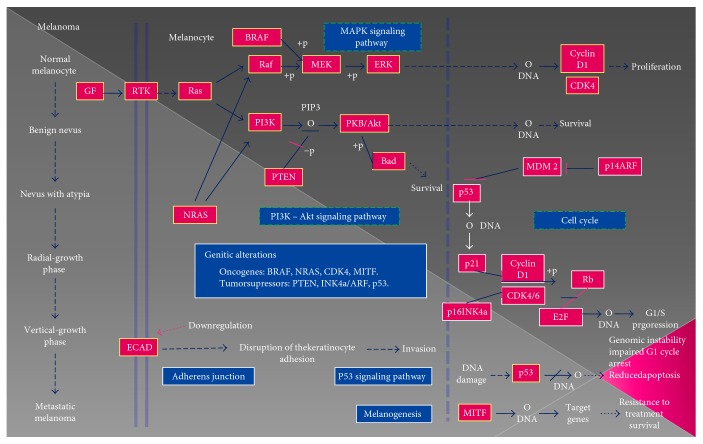
Schematic of the multitude of interacting genetic factors that influence the pathogenesis of melanoma. Oncogenic *NRAS* mutations activate the effector pathways PI3K-AKT and Raf-MEK-ERK. The latter pathway is also activated by means of mutations in the *BRAF* gene. In contrast, PI3K-AKT pathway activation is conditioned by the loss or mutation of the tumor suppressor gene *PTEN*. These changes are generally preserved throughout tumor progression. The development of melanoma has been shown to be strongly associated with the inactivation of the tumor suppressors p16INK4a/cyclin-dependent protein kinases 4 and 6/retinoblastoma (p16INK4a/CDK4,6/pRb) and p14ARF/human double minute 2/p53 (p14ARF/HMD2/P53). Other factors such as microphthalmia-associated transcription factor (MITF) and TP53 play a crucial role in the progression of melanoma [4].

Human melanomas are malignant tumors formed from melanocytes. As an aggressive type of skin cancer, melanoma is a major cause of morbidity and mortality. Notably, the incidence of melanoma is increasing worldwide, and no satisfactory treatments are currently available, with the exception of surgery [1–3]. The development of a melanoma is a dynamic process whereby the immune system not only protects against cancer development but also shapes the characteristics of the emerging tumors through a so-called “cancer-immunoediting” process. Accordingly, new immunotherapies are being investigated to identify and characterize the different subsets of cancer cells in melanoma in order to design individualized treatments for patients. Moreover, metastasis (Figure 1) [4] is a highly complex process, and its mechanisms have been difficult to elucidate in detail owing to the high genetic heterogeneity; nevertheless, the metastasis process is generally associated with severe immune tolerance, which is explained in part by the low percentages of tumor peptides or the poor immunogenicity of melanoma antigens [5]. To select the most effective therapy, the Cancer Genome Atlas Research (TCGA) network divided melanoma into four subtypes based on the presence of mutations in the *BRAF*, *RAS*, and neurofibromatosis type 1 (*NF1*) genes. Therefore, drugs targeting these genes have been developed as candidate treatments for melanoma. However, some tumors show resistance to BRAF inhibitor treatments, and some mechanisms for this drug resistance have been identified to contribute to treatment failure, which are caused by mutations in several genes in most cases [6, 7]. Accordingly, a promising treatment approach for patients with melanoma is combination therapy to simultaneously inhibit multiple pathways, including the BRAF (using vemurafenib or dabrafenib) and mitogen-activated protein kinase (MEK; using trametinib and cobemetinib) pathways, which produces a response in the majority of patients. Moreover, other agents that target the immune system are being actively investigated to improve the efficacy and reduce the toxicity of therapies to cure melanoma, such as the use of anticytotoxic T-lymphocyte-associated protein 4 (CTLA-4) antibodies (ipilimumab) and T cell immunoglobulin and mucin domain 3 (TIM3)/CD137 [8].

Several assays have been performed to validate interferon-alpha (IFN-*α*) as an adjuvant in immunotherapy treatments against resected melanomas. Mocelli et al. [9] conducted a meta-analysis including the results of 14 trials on the use of IFN-*α* as an adjuvant and found a 12% reduction in the risk of death; however, only one study using high doses of IFN-*α* established a significant impact of the treatment on overall survival. Clinical trials are currently underway in patients with *BRAF* mutation-associated melanoma by combining a BRAF-1 and IFN-*α* inhibitor to determine whether the combination might have greater potency than monotherapy.

Nanda et al. [10] reported that the phosphatidylinositol 3-kinase (PI3K)/AKT/mechanistic target of rapamycin (mTOR) signaling pathway is an important regulator of key cellular processes in melanoma and is therefore a candidate combinatorial partner for both targeted and immune therapies.

Neuroblastoma RAS viral oncogene (*NRAS*) mutations are present in 15–20% of all melanomas and are associated with a poor prognosis; the combination of a MEK inhibitor and CDK4 inhibitors has shown promising results in these patients [7]. Loss of or mutations in the phosphatase and tensin (*PTEN*) tumor suppressor gene and *NRAS* mutations are the most common mechanisms through which the PI3K/AKT pathway is activated, thereby mediating cell proliferation, motility, angiogenesis, apoptosis, and metabolism in cancer. In fact, the loss of PTEN expression is predictive of brain metastasis in cases of BRAF-V600 mutant melanoma [7, 11].

Many studies have investigated the relationships among cancer cells, the tumor microenvironment, and the immune system. However, not all therapies that block inhibitory control points of the immune system are effective in all patients. Thus, future investigations are needed to combine these immunotherapies with others that stimulate the immune system at different points to develop personalized treatments for patients based on the specific antigens expressed by their tumor cells. In this review, we provide a comprehensive discussion of the current immunotherapy techniques and offer perspectives for the future in this field.

## 2. Literature Search Strategy

We carried out a comprehensive search of the Cochrane Central Register of Controlled Trials, MEDLINE (PubMed), and Embase databases for articles published from March 2010 to March 2016 using the following search terms: melanoma along with immunotherapy, peptide vaccine, viral vaccine, dendritic cell vaccine, T cell, and/or biochemotherapy. We performed an exhaustive review of published articles and the bibliographies of selected manuscripts.

## 3. Immunotherapy Approaches

Immunotherapy has great potential to promote the development of and progress in the treatment of patients with melanoma. Indeed, recent findings and emerging studies on therapeutic interventions have demonstrated a complete treatment response in specific patient subgroups.

**Figure 2:**
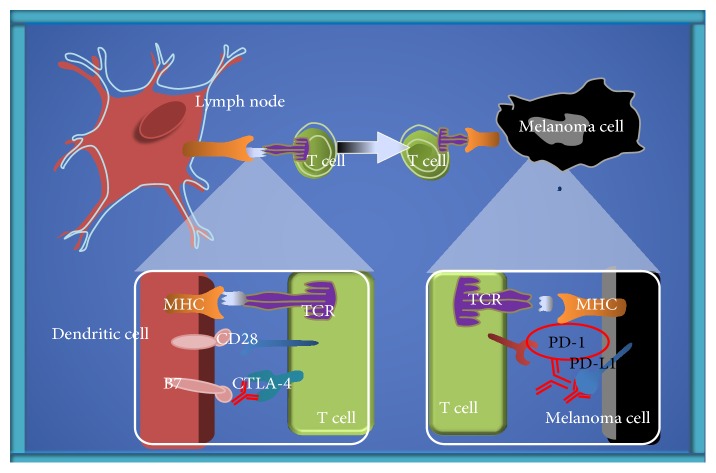
Control of checkpoint blockade and targeted therapy in metastatic melanoma. Four steps are required to attack the tumor cell by the immune system: recognition, tumor antigen presentation to T cells, T cell activation, and direct tumor attack. MHC: major histocompatibility complex; TCR: T cell receptor; CTLA-4: cytotoxic T-lymphocyte antigen 4.

As discussed by Farkona et al. [12], major advances in targeting the immune evasion phase of tumors have been obtained using drugs that block the inhibitory control points that regulate the immune system, such as programmed death 1 (PD-1) and CTLA-4. Several therapeutic targets at immune system checkpoints are under active investigation for drug development. For example, some immunological treatments involving CTLA-4 or PD-1/programmed death ligand 1 (PD-L1) receptors have shown good results in melanoma [13] (Figure 2). Alternatively, OX40 (a T cell stimulator) is a tumor necrosis factor receptor (TNFR) that is associated with increased T cell expansion, proliferation, survival, and memory development. In some cases, OX40 ligation has been shown to suppress the tumor suppressor activity of FoxP3^+^ CD25^+^ CD4^+^ regulatory T cells (Tregs). This suggests that the addition of OX40 stimulation may help to increase the efficacy of dual blockade strategies [14].

Extraction of T cells from patients and the subsequent genetic modification of these cells with chimeric antigen receptors are under development as a promising alternative approach. Contreras et al. [15] inoculated B16F10 melanoma cells that express very low levels of the lymphocytic choriomeningitis virus peptide GP33 (B16GP33) into syngeneic C57BL/6 mice. Subsequently, bona fide, naïve, effector, or memory phenotype GP33-specific CD8^+^ T cells were adoptively transferred into the mice after inoculation. Only the mice that received memory T cell-based adoptive cell transfer (ACT) immunotherapy showed specific durable immunity to melanoma. The authors concluded that the use of nonexpanded CD8^+^ T cells could improve the immunotherapeutic efficacy of ACT.

In addition, the mechanisms of immunotherapies that target tumors can begin to be elucidated based on the results of several recent clinical trials. For example, Redeker and Arens [16] reported the effectiveness of ACT employing tumor-infiltrating lymphocytes (TILs) plus interleukin- (IL-) 2 after lymphodepletion in the tumor. The recent success of ACT strategies suggests that T cell-specific ACT may be more effective in the context of reinfusion after lympho/myeloablative therapy. Alternatively, restimulation of injected TILs with a tumor vaccine using the same antigen that is recognized by TILs may improve the lifespan of antigen-specific T cells [16].

Albeituni et al. [17] further suggested that myeloid-derived suppressor cells (MDSCs) may be a new target of immunotherapy in melanoma since the number of monocytic CD14^+^ HLA-DR-MDSCs is increased in patients with melanoma. In addition, different cellular activities such as suppression of T cell proliferation and natural killer (NK) cell activity, as well as IFN-*γ* production, can impair the quality of dendritic cells (DCs).

Moreover, Chen et al. [18] showed that a natural mycelial polysaccharide of the marine fungus *Phoma herbarum* sp. YS4108, termed YCP, had antitumor effects and could enhance the host immune response. In particular, YCP exhibited a specific immunomodulatory capacity that was mediated by T cells and DCs. Evaluation of the T cell/DC activation-related factors, including IFN-*γ*, IL-12, and IL-4, showed that toll-like receptor- (TLR-) 4 is responsible for the YCP-induced activation in DCs, whereas TLR2 and TLR4 were responsible for the YCP-induced T cell activation.

## 4. Vaccines

With increasing insight into the role of the immune system in melanoma development and progression, vaccines for melanoma are actively being investigated. Several strategies have been used to establish an effective vaccine for melanoma, which fall under the following four main categories: those targeting melanoma cells directly, DC-based vaccines, peptide-based vaccines, and vector-based vaccines. However, most are currently in the testing phase, and promising results have not yet been obtained.

For example, vaccines based on autologous/allogeneic peptides such as canvaxin have not shown good results in phase II studies in patients with stage III and IV melanoma. Dany et al. [19] reported the development of vaccines based on glycolipids such as GM2; however, these vaccines did not improve the clinical response to melanoma. One vaccine that has shown interesting results is based on the tumor antigen gp100. When combined with IL-2 treatment, this gp100 vaccine resulted in increased survival rates. In addition, vaccines using cancer-causing viruses, such as the T-VEC vaccine, have shown increased response rates in phase II clinical trials in melanoma. Tumor-associated antigens represent another potential type of immunization strategy. Tumor-associated antigens are made from specific tumor antigens from cells that are isolated or produced by chemical or genetic synthesis in melanoma [20].

The most relevant studies on vaccine development for melanoma are summarized in Table 1, and each of the four categories is described in detail in the following sections.

### 4.1. Vaccines Targeting Melanoma Cells

Vaccines targeting melanoma cell tumors are a form of active, specific immunotherapy involving the use of parts of melanoma cells or melanoma cells from newly resected tumors obtained during surgery. The tumor cells may originate from the patient, another donor, or several donors.

Giampietri et al. [21] demonstrated that the endoplasmic reticulum (ER) is an important cell organelle involved in several cancer-related processes. In solid tumor cells, the functions of the ER are altered (ER stress) and mechanisms known collectively as the unfolded protein response (UPR) are activated. The UPR activates certain signaling pathways that are responsible for adjusting the tumor microenvironment, which contributes to the resistance to therapies. Factors such as hypoxia, oxidative stress, inflammatory stress, acidosis, nutrient deprivation, and angiogenic growth factors produce ER stress and thus UPR activation, leading to activation of a signaling cascade that contributes to the creation of the tumor microenvironment and the resistance of tumor cells to treatments. The ER chaperone BiP is associated with resistance to various anticancer therapies and is expressed in high amounts in melanoma. Other types of ER chaperones such as XBP1 and ATF6 show increased expression levels in other types of tumors, making them candidate targets for the development of anticancer therapies. One of the key defense mechanisms of the cell against ER stress is the removal of poorly folded proteins through a mechanism of degradation in the proteasome, termed ER-associated protein degradation. SEL1L is a protein involved in this process, and its levels have been correlated with prognosis in several types of cancers [21].

One strategy for the induction of antitumor lymphocytes is the use of vaccines. However, most of the vaccines tested to date have been based on proteins or cells that are more effective as antigens and stimulate the production of CD4^+^ cells rather than CD8^+^ cells. The T cell surface protein 4-1BB is a member of the TNFR family and has been tested for its efficacy in vaccine development [22]. In particular, a melanoma cell line (M20) was transfected with HLA-A2 (A2) and then with a plasmid-encoding 4-1BBL (BBL) and was used for the development of a vaccine. ThisM20/A2/BBL vaccine was shown to increase IFN-*γ* production by 4–6 times that of an A2-transfected M20 (M20/A2) cell line, indicating that the BBL protein is a potent activator of the immune response through CD8 lymphocytes. Notably, no serious side effects were detected. However, 75% of the patients who were monitored for immune responses showed a significant increase in the percentage of IFN-*γ*-producing CD8 T cells and CD107a-expressing CD8 T cells, a marker of cytotoxic activity [22].

Moreover, differences in inflammatory infiltration and production of T helper (Th) 1 cytokines in the vaccine-draining lymph node, with an increase in the antigen-specific effector/regulatory T cell ratio in the lymphoid organs, have been shown to have functional antitumor effects after the application of tumor vaccines such as CpG and poly IC [5]. Zeng et al. [23]demonstrated that the T cell-mediated immune response starts in the lymphoid organs, which are the main targets of cancer vaccines; however, it remains a major challenge to achieve efficient delivery of an antigen/adjuvant at these sites. The authors developed particles that could protect antigens from degradation and prolong their contact with the immune system using encapsulated or conjugated vaccine antigens. The importance of the particle surface characteristics was particularly evident, including smaller hybrid micelles (HMs; 20–200 nm) that can migrate more efficiently to the lymph nodes and the use of PEG-PE and cationic PSA mixed at a 1 : 1 molar ratio to generate hybrid micelles (HM50) that can target the draining lymph nodes and promote the uptake of their cargos by DCs, with a lower risk of systemic toxicity, resulting in significantly greater CTL activity and reduced melanoma growth and metastasis. The authors successfully encapsulated the melanoma antigen peptide Trp2 and the TLR-9 agonist CpG ODN into HMs and demonstrated the high therapeutic potential for this hybrid.

The use of cells as therapeutic agents for cancer treatment is well established with viable cells such as adoptive T cell therapy; however, Ahmed et al. [24] also showed that the use of irradiated, and therefore dying, cells targeting the whole tumor showed promising outcomes to generate effective adaptive immune responses and reduce the possibility of immune evasion by the tumor cells. Cell surface engineering using biomolecules to decorate the surfaces of tumor cells can further enhance the immunogenicity of a vaccine with the possibility of loading a cell particle hybrid into a variety or combination of immune adjuvants, which demonstrated statistically enhanced survival and maximized changes in immune protection and tumor regression [25]. However, suitable models are needed for careful selection of the cellular component prior to their clinical application.

Dai et al. [26] reported that overexpression of the human tyrosine kinase receptor ErbB-2 (HER-2) is closely related to poor prognosis as well as cancer cell migration and invasion, highlighting this molecule as a critical target for cancer immunotherapy. Passive antibody therapies have been shown to improve the outcome of patients with HER-2-positive cancers but tend to result in higher rates of resistance compared to vaccination treatment, thereby requiring a high administration frequency and high costs. Since there were no suitable metastasis models to evaluate the antimetastatic efficacy of HER-2 vaccines, given that an artificial metastasis model cannot simulate spontaneous tumor cell metastasis, the authors developed HER-2-positive murine melanoma B16BL6/E2 cell lines that could metastasize both in vivo and in vitro. Thus, they obtained a stable tumor model that can be successfully utilized in the evaluation of a vaccine's effectiveness in preventing HER-2-positive cancer metastasis and recurrence.

### 4.2. DC-Based Vaccines

**Figure 3:**
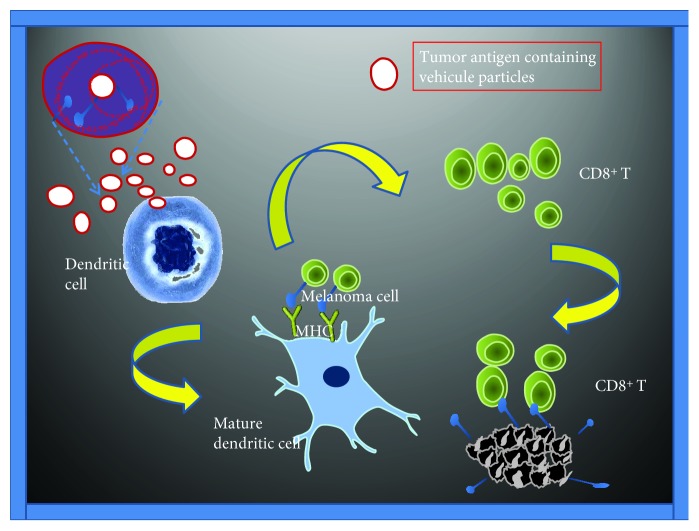
Different vehicles could bind to tumor antigens and adjuvants resulting in antigen-presenting to dendritic cells (DCs). Once these vehicles are absorbed, both the antigen and the adjuvant will be released and degraded, leading to acceleration of the maturation of DCs as well as MHC molecules located on the cell surface that present the antigen. This will allow binding to CD8^+^ T cells that are activated, proliferated, and generated an antitumor response. MC: major histocompatibility complex; TCR: T cell receptor.

DCs are potent and effective antigen-presenting cells and have a high capacity to induce T cell immunity through induction of proinflammatory cytokine responses and stimulation of cytotoxic T cell responses. However, DC-based vaccines are still not fully effective since tumors tend to reside in immunosuppressive microenvironments that reduce their effectiveness. Verma et al. [27] used DCs as vaccines to increase host resistance in patients with melanoma. The DCs were embedded in a matrix of fibrinogen and thrombin (beDCs) and showed elevated IFN-*γ* production when activated by tumor-associated antigens and cytokines. Moreover, mice inoculated with beDCs showed a greater tumor reduction response than those inoculated with free DCs; however, no antitumor activity was observed in tests with a single dose of beDCs. Moreover, when partial resection of solid tumors was performed followed by inoculation of the resected space with beDCs, more than 65% of the mice showed complete remission, and the remaining mice showed a significant delay in tumor growth. In addition, 50% of the mice showing complete remission developed an effective immune response against reintroduced tumor cells of the same type. Thus, these DC matrices were able to interact with host immune system cells and to attract lymphocytes (Figure 3).

This interaction then allows for the development of tertiary lymphoid structures that are associated with positive responses to immune therapies. Accordingly, the use of beDC-based immune therapies combined with other substances such as certain drugs or cytokines could help in the development of effective immune responses to various types of tumors.

**Figure 4:**
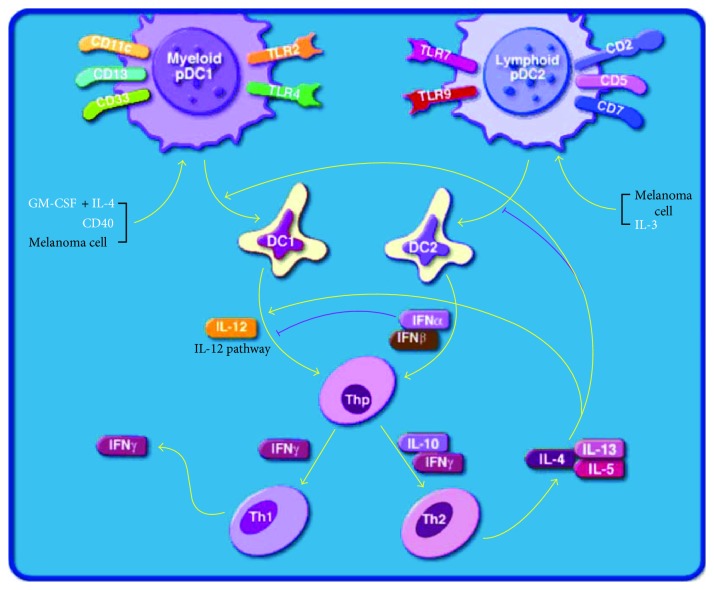
Dendritic cells regulating Th1 and Th2 development in melanoma (modified from the BioCarta database) [29].

Bronchalo-Vicente et al. [28] highlighted the adjuvant properties of listeriolysin O (LLO) (a hemolysin secreted by *Listeria monocytogenes*, the pathogen that causes listeriosis), such as activation of DCs, stimulation of potent cytotoxic T cells disrupting the immune response to tumors, and enhancement of Th1-dominant immune responses (Figure 4) [29].

Moreover, LLO has been shown to target melanoma cells and transform these cells into DCs, resulting in melanoma regression. The immune-dominant response of LLO peptide (amino acids 91–99) when presented to cytotoxic T cells, thereby affecting the immune response to other antigens, is relevant for the development of cancer and prophylactic vaccines. Moreover, immunotherapy with LLO incorporated into a DC vaccine has been shown to prevent the adhesion, dissemination, and metastasis of B16OVA melanoma cells and could induce robust innate and specific immune responses to *Listeria* infection and melanoma [30]. LLO induces effector CD8^+^ T cells that are localized within the tumor and show efficient adjuvant properties. *Listeria* vaccines induce strong cytotoxic T cell responses, and vaccination with such vaccines has been shown to elicit a 20-fold reduction in the mitotic index of the melanoma cells. Furthermore, DC-LLO eradicates melanoma by programmed cell death through induction of a 2.6-fold increase in early apoptosis [30]. In addition, a high percentage of NK cells of the tumorigenic phenotype CD3^−^CD49b^+^ and phenotypes involved in tumor elimination, for example, CD8^+^CD11c^+^CD83^+^CD86^+^iNOS^+^MHC-II and DC-Lcd11b^+^ macrophages, was observed, along with high levels of IFN-*γ* and IL-12 Th1 cytokines and the percentages of CD8^+^ cells. The percentages of Tregs showing the phenotype CD4^+^CD25 high FoxP3^+^ were also decreased in the TILs of melanoma after administration of the DC-LLO vaccine [30]. In summary, DC-LLO vaccinations promote stronger antimelanoma immune responses, increase the positive signals between DCs and T cells, and control tumor growth and dissemination [28, 30].

### 4.3. Peptide Vaccines

Iversen [31] conducted a preliminary phase II study of a combined treatment with a vaccine (IDO + survivin peptides, combined with montanide, imiquimod, and granulocyte macrophage colony-stimulating factor (GM-CSF) as adjuvants) plus the chemotherapeutic agent temozolomide (TMZ). Of the 31 enrolled patients with stage IV melanoma, 15 were screened to be positive for HLA-A2 expression. The preliminary clinical data of seven patients showed that two had partial remission, three had stable disease for 3 months or more, and two showed disease progression with the appearance of new lesions.

In addition, Hu et al. [32] vaccinated patients with stage IV melanoma with six melanoma helper peptides (6MHP). The overall 1- and 5-year survival rates were higher in 38% and 41% of the vaccinated patients, respectively, compared with those in the matched control groups. Moreover, 65% of the vaccinated patients developed a specific immune response to 6MHP in the peripheral blood, and their 1- and 5-year survival rates were 28% and 24% higher, respectively, than those in patients showing no immune responses. The results of this study were superior to those of previous studies testing other treatments in patients with stage IV melanoma.

Reed et al. [33] immunized patients with a peptide vaccine at different doses. At week 7, 23 of 37 patients showed both antibody and T cell responses, four patients showed no response, two patients showed an antibody response only, and five patients showed a T cell response only. Of the six peptides included in the vaccine, the three longest peptides (FLL: tyrosinase_386–406_; RNG: MART-1/Melan-A_51–73_; and WNR: gp100_44–59_) showed better induction of immune responses. Peptide length was well correlated with the antibody response (*R* ^2^ = 0.82).

Checkpoint inhibitors show great potential for the development of antitumoral therapies. However, these therapies are associated with some problems such as toxicity and resistance. The PRAME tumor antigen is one of the targets of cytolytic T lymphocytes and is expressed in multiple tumors, including metastatic melanoma tumors. Gutzmer et al. [34] conducted a phase I study to evaluate the dose-related toxicity and humoral immune response of anti-PRAME therapy, including 66 patients with stage IV M1b-c melanoma who were divided into three groups that received a different dose of the immunotherapy (RECPRAME + AS15). In all three groups, the associated adverse effects and toxicity were maintained to a clinically acceptable extent. Immunotherapy induced a humoral immune response as well as a CD4^+^ T cell response to PRAME. CD4^+^ T cells are responsible for promoting the functions of CD8^+^ cells and favor the elimination of tumor cells. Although this was only a phase I study, the results obtained demonstrate that PRAME may be an effective target in antitumor therapy.

**Figure 5:**
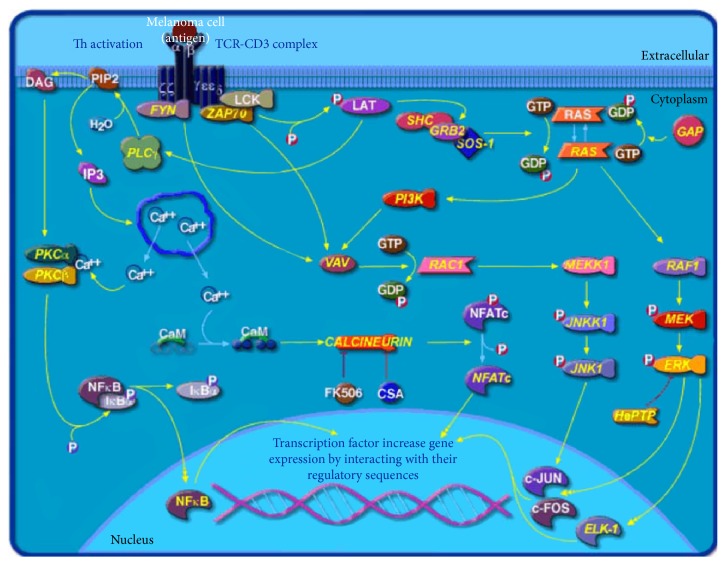
T cell receptor (TCR) signaling pathway in melanoma (modified from the BioCarta database) [29].

Kumai et al. [35] attempted to develop a vaccine that generates a significant amount of antigen-specific CD4 helper T lymphocytes (HTLs). The mice were divided into several groups and administered intravenously with a vaccine (TriVax) containing a peptide, a CD40 monoclonal antibody (CD40mAb), and different TRLs (Figure 5) [29].

First, the mice were inoculated with B16F10 cells to induce tumor formation (melanoma and lymphoma). After 3–10 days, the mice were injected with a first dose of the vaccine, and a second dose (boost) was administered at 12 days. The vaccine was shown to induce a potent CTL response and also produced a more potent CD4 T cell response to 2W1S peptide compared to a peptide + lipopolysaccharide-based vaccine. Furthermore, the addition of OX-40 (an agonist monoclonal antibody) to TriVax improved the CD4 T cell response to the OVA peptide. The ligands TLR5 and TLR7 are known to stimulate the HTL response more effectively than the TLR3 ligand. Four TLR agonists were evaluated as TriVax/OX40 adjuvants, and gardiquimod, a TLR7 agonist, was shown to induce the most potent CD4 T cell response.

### 4.4. Vector-Based Vaccines

Viruses have the ability to infect cells and can stimulate an immune response. Vaccine viruses (VVs) have been widely used as gene therapy vectors, acting as oncolytic agents because of their ability to activate the immune system against tumors via the production of cytokines or other immunomodulatory molecules. Moreover, VV scan evades the host immune response.

DNA-dependent activator of interferon regulatory factor (DAI), also known as Z-DNA-binding protein 1 (ZBP1) or DLM-1, is a cytosolic double-stranded DNA sensor that strongly activates the innate immune response. Thus, DAI may be used as a genetic vaccine against melanoma and stimulus-specific effector T cells of the tumor. Indeed, Hirvinen et al. [36] showed that DAI-expressing plasmids could more efficiently induce memory and effector tumor-specific T cells; similarly, myeloid differentiation factor 88 (MyD88) was shown to potentiate systemic antitumor immunity. Thus, the oncolytic VV expressing DAI boosts the innate immune system and activates immune cells in the tumor. Accordingly, infection with DAI-expressing VVs promotes the upregulation of several genes in monocytes related to important immunological pathways. In addition, DAI expression by oncolytic vaccines was shown to enhance cancer eradication in vivo by inducing antitumor T cell responses in a mouse model. In the same study, an oncolytic Western Reserve strain vaccinia virus specific to epidermal growth factor receptor pathway mutations (vaccinia growth factor) and tumor-associated hypermetabolism (thymidine kinase) was conjugated with human or murine DAI and a tdTomato fluorophore, and the concentration of infectious virus particles was determined by a standard crystal violet staining assay with A549 cells. The authors concluded that the antiviral T cell responses were less prominent than antitumor T cell responses and that the ability of the DAI-armed vaccinia virus as a self-sensing and immuno-boosting system to change the immunosuppressive tumor microenvironment would be of great help in developing new vaccination strategies [36].

One type of immunotherapy used to stimulate the immune system against tumors is in situ vaccination. For this purpose, oncolytic viruses are injected directly into the tumor or metastasis zone, thereby reducing the risk of side effects. In one study [37], mice with metastatic lung melanoma and other types of tumors were vaccinated in situ with empty *Cowpea mosaic virus* (eCPMV); necrotic centers formed in the tumors, resulting in complete removal of the tumor in half of the mice after only two vaccinations. The use of eCPMV was more effective than vaccination in situ with other immunogenic compounds. In addition, the mice acquired a protective systemic immune response against induction of the same tumor, which was rejected. The immune response triggered by eCPMV requires Th1- and IFN-*γ*-associated IL-2 adaptive immunity and neutrophils, favoring the existing and/or new antitumor immune response. There were no increases in the levels of proinflammatory cytokines (tumor necrosis factor-alpha and IL-6), which cause tissue damage in the lung. Moreover, in situ vaccination with eCPMV is well tolerated and does not result in observable side effects. Indeed, in situ vaccination with eCPMV also showed very promising results in an ovarian carcinoma model and in two models of metastatic cancer of the colon and breast. Thus, eCPMV is not only limited to serving as an in situ vaccine but also may have applications as a carrier for various immune adjuvants, further enhancing the immune response to tumors.

Because of the increasing incidence of malignant melanoma, the development of different therapeutic agents to improve the prognosis for melanoma patients is a primary target. GM-CSF enhances immune responses through the stimulation of DCs and B and T lymphocytes and the recruitment of NK cells. By contrast, TGF-B2 produced by cancer cells represses the immune response and improves the development of tumor cells, indicating that TGF-B blockade is indispensable to an effective immunotherapeutic strategy. In addition, oncolytic viral infection of tumor cells induces antitumor immune responses. Based on this background, Kim et al. [38] studied the administration of a complex form of a DNA vaccine (GM-CSF, small hairpin RNA against TGF-B, and MART1) and an armed oncolytic adenovirus and found that the combined treatment induced the greatest antitumor effect in an immunocompetent mouse model system when compared with individual treatments.

### 4.5. Other Vaccine Types and Prospects

DNA vaccines offer advantages as antitumor therapies, and their safety and immunogenicity have been demonstrated in clinical trials; however, these vaccines have not shown great effectiveness. Gordy et al. [39] tested a vaccine containing a DNA plasmid encoding the chemokine MIP3*α* and the gp100 antigen in the mouse melanoma cell line B16F10. The mice were vaccinated three times at 1-week intervals, and the results were evaluated against vaccines containing only the antigen or placebo vaccines. The results showed that the MIP3*α*-gp100-based vaccine caused greater elevation in the antibodies to B16F10 compared to the two other vaccines tested. In addition, the tumor growth rate slowed down and mouse survival improved. This vaccine activates both CD8^+^ and CD4^+^ effector T cells. This study showed that the addition of MIP3*α* to therapeutic vaccines could serve as an adjuvant to achieve better immunogenicity and improve the immune system response to tumors.

Other newly identified candidate vaccine agents are in clinical development. For example, allovectin-7 (a plasmid/lipid complex with DNA sequences encoding HLA-B7 and B2 microglobulin) induces a 5-fold increase in the frequency of HLA-B7 cytotoxic T cells, upregulates/restores MHC-1 molecules, and induces a proinflammatory response. OncoVEX (oncolytic herpes simplex virus encoding GM-CFS) has the ability to replicate selectively in tumor cells, and local expression of GM-CFS is thought to be synergic. Finally, PV-10 (a small-molecule fluorescent derivative), which is selectively taken up by the plasmalemma of cancer cells and accumulates in the lysosomes, triggers lysosomal release and leads to autolysis. These agents, along with other combinations, are being pursued by different investigators [3, 40].

## 5. Biochemotherapy

Biochemotherapy is the use of immunotherapy in combination with chemotherapy. Several clinical trials have evaluated the effectiveness of adjuvant biochemotherapy for the treatment of high-risk melanoma and as a unique treatment for advanced melanoma.

One of the major challenges in realizing effective immunotherapies against cancer is overcoming the microenvironment that is generated in tumors. In this microenvironment, in addition to malignant cells, there is a heterogeneous group of other cell types (fibroblasts, immune system cells, and endothelial cells) and several molecules secreted by tumor cells such as growth factors and cytokines. Notably, sunitinib, a tyrosine kinase receptor inhibitor, has direct effects on inhibiting tumor growth by promoting apoptosis and inhibiting the induction of endothelial growth factor by tumor cells. In addition, sunitinib may inhibit tumor growth in an indirect manner by stimulating the antitumor immune response. One study [41] demonstrated the efficacy of a tumor-specific antigen-based vaccine embedded in a mannose-modified lipid calcium phosphate (LCP-Trp2) nanoparticle on the regression of induced melanoma in mice. However, this efficacy only occurred in the early stages of tumor growth (4 days after tumor inoculation) and not in the late stages (13 days after tumor inoculation) despite similar CTL responses in both cases. Indeed, inhibition of tumor growth was observed with sunitinib administered intravenously in an oral suspension or encapsulated in micelles, and the effects of the latter were further enhanced by coadministration of the LCP-Trp2 vaccine; this combined treatment increased the induction of apoptosis in tumor cells.

When used in combination with drugs, vaccines stimulate strong immune responses against the specific targets; these responses can inhibit the immune suppression of T cells to optimize immunotherapy in metastatic melanoma. Moreover, this combination enhances the level of tumor-infiltrated CD8^+^ T cells and the CD8/Treg ratio in the tumor microenvironment, thereby promoting tumor rejection [8].

As additional combinations of chemotherapies and molecular-targeted treatment agents with melanoma vaccines, researchers have evaluated the use of vaccines combined with cyclophosphamide to enhance antigen-specific immune responses. Notably, it is important to determine the optimal scheduling of various immunomodulators in combination therapies [8]. Alternatively, combinations such as radiotherapy plus immunotherapy have been shown to have proimmunogenic effects because the synergistic effects of ionizing radiation can stimulate antitumor immunity (i.e., convert tumors that are refractory to immune checkpoint inhibitors into responsive tumors) by generating an in situ vaccine. Such treatments have also been shown to induce systemic responses (abscopal effects) [7, 8].

Because localized and systemic chemotherapies have been used based on tumor localization/stage and metastasis, recent studies have sought to determine the effects of the receptor for the lipid mediator PAF (PAFR), a G-protein expressed in several cell types. PAFR acts as an agonist in systemic chemotherapy performed in cell models in vitro. Moreover, Sahu et al. [42] demonstrated that systemic chemotherapy with etoposide decreased the growth of melanoma prior to the implantation of tumor cells. Thus, PAFR agonists may alter the effects of systemic chemotherapy.

Cui et al. [43] provided a comprehensive review of the outcomes of clinical trials using a combination of chemotherapy and biochemotherapy. Overall, these treatments showed poor efficiency; however, when used in combination with antivascular endothelial growth factor (VEGF) antibodies, the therapeutic effects were significantly increased. For example, in a phase II trial in patients with metastatic melanoma treated with paclitaxel/carboplatin (PC) or with PC plus bevacizumab (CPB), the median progression-free survival was increased in patients treated with CPB compared to the placebo control, with relative risks of 25.5% and 16.4%, respectively. However, patients with mucosal melanoma treated with CPB showed a 76% reduction in the risk of death. In a phase II trial of patients with metastatic melanoma treated with TMZ combined with bevacizumab, the overall survival was 12 months in patients with wild-type *BRAF* but was only 9.2 months in patients with mutated *BRAF*. This suggests that chemotherapies combined with antiangiogenic therapies may be more effective in patients without *BRAF* mutations. Tyrosine kinase inhibitors are small molecules that inhibit the VEGF and platelet-derived growth factor receptor pathways. In another study of 37 Chinese patients with metastatic melanoma, the combination of TMZ, bevacizumab, and the tyrosine kinase inhibitor sorafenib was found to be more effective than traditional chemotherapeutic treatments. Greater efficacy of paclitaxel with the tyrosine kinase inhibitor pazopanib was also observed in a phase II study in patients with stage III and IV inoperable melanomas [43].

Other immunotherapy treatments in cutaneous melanoma involve the use of ipilimumab in combination with electrochemotherapy (ECT) to open the pores in tumor cells so that antitumor agents can enter with ease. The advantage of ECT is its safety and few side effects. Theurich et al. [44] evaluated clinical data including 127 consecutively treated melanoma patients at four cancer centers in Germany and Switzerland that received either ipilimumab (*n* = 82) or ipilimumab and additional local peripheral treatments, including ECT or radiotherapy (*n* = 45), if indicated for local tumor control. Patients that received the combination of local treatments plus ipilimumab showed significantly increased overall survival compared to those treated with ipilimumab alone (93 versus 42 weeks).

In another retrospective multicenter study, Heppt et al. [45] evaluated the effects of the combination of local ECT plus ipilimumab or PD-1 inhibition in a total of 33 patients from 13 centers with a median follow-up time of 9 months. Patients treated with ipilimumab plus ECT did not reach the median overall survival, whereas patients in the PD-1 group had an overall survival of 15 months. These findings highlight the potential benefit of the combination of ipilimumab with ECT.

Finally, Franzese et al. [46] reported that Melan-A-specific CD8 cells isolated from long-term surviving patients treated with dacarbazine before peptide vaccination plus IFN-*α* exhibited higher antitumor reactivity and an enlarged T cell repertoire compared with those of patients treated with the vaccine alone. These data suggested that the phenotypic and functional T cell signature elicited by chemoimmunotherapy is a fine-tuned balance between the quality of AKT activation and antitumor T cells, which can help to protect patients from tumor recurrence.

## 6. Conclusion

Advances in immuno-oncology have improved our understanding of the interaction between the immune system, cancer cells, and the tumor microenvironment, and application of these discoveries has great significance for the treatment of melanoma. Treatments based on molecular inhibitors are only effective in some patients. However, rational combinations between new immunotherapy technologies and immunotherapeutic agents are currently being evaluated. In addition, tumors can create antigens and neoantigens that are very different from those of normal tissues; thus, elucidating these differences may be the key for developing customized immunological strategies with decreased side effects and increased immune responses. One important field to develop is prophylactic vaccination for the prevention of melanoma. In the foreseeable future, prophylactic vaccines will probably only be used to a limited extent. Thus, current research efforts are primarily aimed at the development of therapeutic vaccines that can reduce the tumor volume or provide protection against relapse in patients who have already had cancer. The ultimate goal is to achieve effective therapies for metastatic disease. Moreover, the presence of tumor-infiltrating mononuclear cells (TIMC) and the absence of PD-L1 are reportedly associated with a better response to treatment. Thus, TIM-3, LAG-3, and CEACAM1 are some molecules that can be blocked by monoclonal antibodies. Agents capable of inhibiting immunosuppressive metabolites, such as IDO or arginase, could also be considered for therapy.

Finally, advanced *omics* technologies and computational techniques now provide an opportunity to evaluate not only the genomic variants as they currently are but also the related pathway and network aberrations. This insight will greatly facilitate the selection of drug combinations and thus benefit personalized medicine to improve the quality of life and outcome of patients with melanoma. However, there are still many questions that remain to be tackled in this field, which should be the focus of the future studies. In particular, it is crucial to develop therapeutic strategies that can avoid the potential toxicity of drugs and integrate newly designed biomarkers; such strategies with consideration of immunotherapy are expected to help overcome the challenge of therapeutic resistance in melanoma treatment and other cancers.

## Figures and Tables

**Table 1:** 

Vaccine type	Number of patients	Treatment/drug	Cells used	Murine cell line	Antigen	Administration path	Tumor type	Melanoma type	Murine melanoma cell line	Reference
Vaccines targeting melanoma cells	34	4-1BBL	—	—	—	Upper arm or thigh	Stage IIIB/IV melanoma	SH-M20	—	[22]
Vaccines targeting melanoma cells	NA	Polymeric HMs	—	—	Trp2 peptide	SC	Lung metastatic melanoma	B16F10	C57BL/6	[23]
					TLR-9 agonist	SC				
Vaccines targeting melanoma cells	NA	HSP65-Her-2	—	—	—	Right hind footpads	Lung metastatic melanoma	B16BL6/E2	C57BL/6	[25]
DC-based vaccines	NA	Biomatrices	BMDCs	—	—	SC	Melanoma primary tumor	TC1 or B16	C57BL/6	[26]
						SC near resection	Melanoma postsurgery secondary tumor			
DC-based vaccines	—	BMDCs	BMDCs	C57BL/6	Listeriolysin O peptide	SC	Peritoneal melanoma	A-375/Mel-H0	B16F10	[27]
DC-based vaccines	—	BMDCs	BMDCs	C57BL/6	Listeriolysin O peptide	PC	Liver and lung metastatic melanoma	B16OVA	B16F10	[29]
DC-based vaccines	—	MIP3*α*	—	C57BL/6	gp100	IM (electroporation)	Melanoma	B16F10	C57BL/6	[30]
Treatment	40	TMZ	—	—	—	CT	Metastatic melanoma	—	—	[31]
Treatment	35	IFN-*α*/IL-2			—	IT	Metastatic melanoma			
Peptide-based vaccines	15	IDO peptide			IDO peptide	SC	Stage III/IV NSCLC			
Treatment/peptide-based vaccines	7	TMZ/IDO/survivin peptide			IDO/survivin peptide	CT/SC	Metastatic melanoma			
Peptide-based vaccines	40	6MHP	—	—	gp100/tyrosinase (14aa)/tyrosinase (20aa)/melan-A/MART-1/MAGE-A3/MAGE-A1, 2,3,6	—	Stage IV melanoma	—	—	[32]
Peptide-based vaccines	35	6MHP	—	—	gp100/tyrosinase (14aa)/tyrosinase (20aa)/Melan-A/MART-1/MAGE-A3/MAGE-A1, 2,3,7	—	Stage IIIB/IV melanoma	—	—	[33]
Peptide-based vaccines	66	recPRAME + AS15	—	—	—	IM (deltoid or thigh)	Stage IV M1b-c melanoma	—	—	[34]
Peptide-based vaccines	—	TriVax/OX40 agonist	—	—	OVA/2W1S/VV H3L/Trp1 (14aa)/Trp1 (8aa)/hgp100	IV/IP	Melanoma	B16F10	C57BL/6/B6-Ly5.1	[35]
Vector-based vaccines	—	Vaccinia virus + DAI	—	—	—	—	Human lung cancer/human melanoma/mouse melanoma	A549/HS294T, A2058, SK-MEL-2/B16-F10	—	[36]
Vector-based vaccines	—	eCPMV	—	—	CD45/MHC-II/CD86/CD11b/F4/80/Ly6G/CD16/CD32	In situ	Lung metastatic melanoma/mammary carcinoma	B16F10/4T1-luc/CT26	C57BL/6J	[39]
Vector-based vaccines	—	Oncolytic adenovirus	—	—	MART-1	In situ/IM	Melanoma	B16BL6	C57BL/6	[40]
Vector-based vaccines	—	MS-OVA/LM-OVA	—	—	—	SC	Melanoma	B16F0-OVA	C56BL/6	[41]

6MHP: 6 melanoma helper peptides; BMDCs: bone marrow-derived dendritic cells; CT: chemotherapy; DAI: DNA-dependent activator of IFN-regulatory factors; DTIC: dacarbazine; HMs: hybrid micelles; IM: intramuscular; IP: intraperitoneal; IM: immunotherapy; IV: intravenous; PC: peritoneal cavity; SC: subcutaneous temozolomide.
